# Rethinking the gendered burden of female partners in male factor infertility: a perspective

**DOI:** 10.4069/whn.2025.11.03

**Published:** 2025-12-31

**Authors:** Eleanor L. Stevenson, Jill E. Sergison, Kevin R. McEleny, Eilis Moody

**Affiliations:** 1 Duke University School of Nursing, Durham, NC, USA; 2 Newcastle Upon Tyne Hospitals NHS Foundation Trust, Newcastle upon Tyne, UK

## Introduction

Despite decades of research on infertility, the field continues to overlook how emotional labor is distributed within heterosexual couples—particularly when the infertility diagnosis lies with the male partner. Male factor infertility (MFI) accounts for approximately 30% to 50% of infertility cases worldwide [1]. In heterosexual relationships, this means male partners are increasingly acknowledged in research and counseling protocols. However, the experiences of female partners—who often bear both the procedural demands of treatment and the emotional weight of their partner’s diagnosis—remain insufficiently examined. Research indicates that men and women often process infertility differently, reflecting gendered emotional patterns. This personal view highlights how a diagnosis of MFI can impose additional psychological burdens on female partners and proposes strategies to address the complex emotional needs of couples navigating MFI.

## Male factor infertility and gendered disclosure norms

The psychosocial impact of infertility on men has recently emerged as an area of inquiry. Studies show that MFI may be perceived by men as a threat to masculinity, leading to shame, isolation, and psychological distress [2]. In response, men may suppress their emotions or avoid disclosure entirely, often leaving female partners to navigate the diagnosis in relative silence. Research suggests that when MFI is the cause of infertility, women are significantly less likely to disclose the diagnosis to family and friends, often to protect their partner from perceived stigma [3].

This dynamic illustrates a broader gendered expectation for women to act as emotional gatekeepers. The female partner, though undiagnosed with infertility, may be placed in the position of managing not only her own reproductive goals but also her partner’s emotional well-being. This includes concealing distress, limiting her own support-seeking behaviors, and carefully curating the social narrative of infertility. Such dynamics can be described as hermeneutic labor, a subset of emotional labor in which women are expected to interpret and manage both their own emotional states and those of their partners in intimate relationships [4].

## Emotional labor meets medical burden

This emotional responsibility occurs in tandem with the clinical burden of infertility treatment, which falls disproportionately on women regardless of which partner has been diagnosed. Procedures such as ovulation induction, egg retrieval, and embryo transfer are inherently gendered interventions. As Boivin and Schmidt [5] have argued, the physical invasiveness of treatment coupled with its psychological demands contributes to anxiety, depression, and treatment discontinuation among women.

A cohort study examining why insured women terminated in vitro fertilization found that psychological burden, not cost, was the most commonly cited reason for stopping treatment [6]. This is a striking finding, especially in the context of MFI, where women undergo treatment for physiological issues not their own. The asymmetry is stark: men may face identity-based stigma, but women must process the diagnosis emotionally while undergoing treatment physically.

## Dyadic stress and relational consequences

Infertility affects not only individuals but also relationships. In heterosexual couples, the distribution of emotional labor has implications for relational health. Schmidt et al. [7] found that infertility-related distress can lead to either marital benefit or decline, depending on the couple’s communication strategies and coping mechanisms. According to a review of infertility and marital dynamics, MFI tends to have a more detrimental impact on female partners’ relationship satisfaction, even when men report relatively stable or improved outcomes [8].

This discrepancy may stem from the gendered labor of emotional caretaking. When female partners are expected to reassure, protect, and emotionally stabilize their male partners, while navigating their own grief and uncertainty, the relational balance becomes unsustainable. Lack of mutual support can manifest as increased emotional distance, communication breakdown, and long-term dissatisfaction [9]. Conversely, outcomes tend to be more favorable when couples adopt shared disclosure strategies and foster joint meaning-making [10].

## Clinical and research implications

The implications of these gendered dynamics for clinical practice are significant. First, infertility care must expand beyond the individual diagnosis to address relational processes. Clinical settings may designate the male patient as the “patient of record,” unintentionally marginalizing the psychosocial experiences of his female partner. In cases of MFI, the woman’s emotional burden can be overlooked simply because she is not the source of the diagnosis. This oversight can lead to missed opportunities for intervention and support.

Second, dyadic screening and counseling should be integrated into infertility care models. Healthcare providers should assess how emotional labor, disclosure strategies, and support needs are distributed between partners—not only for the individual undergoing treatment or receiving the diagnosis. Couples-based counseling approaches, which emphasize mutual validation and communication, may mitigate some of the burdens associated with unilateral emotional caregiving.

Third, future research must rigorously explore the experiences of female partners in MFI. Existing studies tend to focus either on infertile men or women, rarely examining how a partner’s diagnosis affects the non-diagnosed individual in gendered ways. Longitudinal, mixed-method studies are needed to investigate how emotional labor evolves and how it influences treatment decisions, mental health outcomes, and relationship satisfaction.

## Conclusion

Infertility is more than a medical condition; it is a complex social, emotional, and relational event. In MFI, the tendency to center the diagnosed individual can obscure the experiences of female partners who are navigating both the physical demands of treatment and the psychological toll of emotional caregiving. These women are not ancillary figures in the infertility narrative—they are active participants whose emotional labor is both profound and unacknowledged.

A reproductive care model that fails to recognize these labor risk reinforce the very gender disparities it ought to dismantle. To move toward equitable and effective infertility care, researchers and clinicians must center the relational and gendered dimensions of emotional burden—not as secondary considerations, but as essential components of health and healing.
